# Combination therapy based on pegylated interferon alfa improves the therapeutic response of patients with chronic hepatitis B who exhibit high levels of hepatitis B e-antigen at 24 weeks: A retrospective observational study

**DOI:** 10.1097/MD.0000000000017022

**Published:** 2019-09-06

**Authors:** Yafei Zhang, Wei Li, Zhongping Liu, Jun Ye, Guizhou Zou, Zhenhua Zhang, Jiabin Li

**Affiliations:** a Department of Infectious Diseases, The First Affiliated Hospital, Anhui Medical University; b Department of Infectious Diseases, The Second Affiliated Hospital, Anhui Medical University, Hefei; c Department of Liver Diseases, Fuyang Second People's Hospital, Fuyang, China.

**Keywords:** biological marker, chronic hepatitis B, combination therapy, interferon, nucleos(t)ide analogues, response prediction

## Abstract

Pegylated interferon alpha (PEG-IFN-α) is a first-line treatment for patients with chronic hepatitis B (CHB), but its efficacy varies from individual to individual. Early discrimination between responder and non-responder patients is important for optimal clinical management. In addition, low therapeutic efficacy is still a major issue; thus, treatment timing should be optimized.

We reviewed our experience with hepatitis B e-antigen (HBeAg)-positive patients treated with PEG-IFN-α, alone or in combination with nucleoside analogues (NAs), from 2009 through 2014. Collected data included both general characteristics of 113 patients and laboratory data at baseline and at treatment weeks 12, 24, 52, and 76. The endpoint was HBeAg seroconversion at week 76.

A total of 113 patients with changed to or start of NAs therapy were included in this study. At the end of treatment, 44 (38.9%) patients exhibited HBeAg seroconversion. Patients with HBeAg seroconversion had lower baseline HBeAg (475.5 vs 751.7; *P* = .007). The incidence of HBeAg seroconversion was significantly higher among patients with HBeAg ≤ 500 signal-to-cutoff ratio (S/CO) (OR = 2.60, 95% CI: 1.16–5.83, *P* = .02) at baseline, HBeAg S/CO ≤ 20 (OR = 3.37, 95% CI: 1.47–7.73, *P* = .003), or a higher than 10-fold HBeAg drop (OR = 3.55, 95% CI: 1.50–8.37, *P* = .003) at week 12 or HBeAg ≤ 15 S/CO (OR = 10.35, 95% CI: 4.09–26.20, *P* < .001) at week 24. Subgroup analyses demonstrated that in patients with HBeAg >20 S/CO at 24 weeks, the addition of NAs treatment may increase HBeAg seroconversion (23.3% vs 0%, *P* = .03).

HBeAg levels had an impact on the rate of serological conversion in CHB patients receiving PEG-IFN-based treatment. Combination therapy with NAs should be considered in CHB patients maintaining a high HBeAg level after 24 weeks of PEG-IFN monotherapy.

## 1 Introduction

Despite the availability of safe and effective vaccines, the global burden of hepatitis B virus (HBV) infection remains high. The World Health Organization estimated that, in 2015, approximately 257 million persons were infected by HBV (defined as hepatitis B surface antigen-positive), and that nearly 1 million persons die each year from HBV-related liver failure, cirrhosis, and hepatocellular carcinoma (HCC).^[1]^ Antiviral therapy reduces the risk of liver disease and the development of HCC and may even contribute to the reversion of liver fibrosis in some patients with chronic hepatitis B (CHB).^[2–5]^ In addition, previous studies have shown that antiviral therapy can reduce disease recurrence and improve survival in patients with HBV-related HCC.^[6,7]^ The primary goal of therapy for chronic HBV infection is to reduce the related complications, particularly the risk of HCC development, and improve the quality of life and the survival of the infected subjects.^[8–10]^

At present, there are 8 medications for CHB treatment, which can be divided into two categories according to the mechanism of action: immunomodulatory agents (interferon alfa, IFN-α, and pegylated interferon alfa, PEG-IFN-α); and nucleos(t)ide analogues (NAs), i.e., the nucleoside analogues lamivudine (LAM), entecavir (ETV), and telbivudine (LDT) and the nucleotide analogues adefovir dipivoxil (ADV), tenofovir disoproxil fumarate (TDF), and tenofovir alafenamide (TAF). NAs effectively inhibit HBV polymerase and are generally well tolerated. However, NAs inhibit HBV replication at a late stage of the viral life cycle and have limited or no effect on viral protein production or the persistence of covalently closed circular DNA, resulting in high rates of disease relapse once NAs are discontinued. Long-term treatment is also limited by the emergence of drug resistance phenomena, potential late adverse effects, cost-effectiveness, mental burden, and durability after treatment cessation.^[11,12]^ By contrast, interferon (IFN, PEG-IFN-α) has both antiviral and immunomodulatory activities and can produce a robust off-treatment response in CHB patients. The main advantages of IFN include a finite treatment duration and higher rates of hepatitis B e-antigen (HBeAg) seroconversion, as well as hepatitis B surface antigen loss (HBsAg), compared to NA treatments of equivalent duration. The major downsides to the use of IFN are the need of subcutaneous injections and the poor tolerability due to multiple adverse effects.^[13,14]^ Notably, currently available monotherapies based on oral NA or IFN administration failed to achieve HBeAg seroconversion and/or HBsAg elimination in most CHB patients within 1 year. The success in the treatment of hepatitis C virus (HCV) and human immunodeficiency virus (HIV) suggests that combination therapies may be an important strategy to improve efficacy and reduce viral breakthrough. Two options are currently available for combination treatment: “NA plus NA” or “NA plus PEG-IFN-α” therapies. Regarding the NA-plus-NA combination, the European Association for the Study of Liver (EASL) recommends that in patients with incomplete suppression of HBV replication, reaching a plateau response during prolonged treatments with ETV or TDF/TAF, a switch to the other drug or to a combination of both drugs may be considered.^[9]^ The combination of NA and PEG-IFN-α can be divided into three categories: initial treatment with NA followed by PEG-IFN-α addition and continuation with NA; initial treatment with PEG-IFN-α followed by addition of NA; simultaneous administration of NA and PEG-IFN-α.^[8]^ Some studies have shown that a PEG-IFN-α/NA combination therapy can improve treatment efficacy, but the optimal drug addiction timing is controversial.^[15–17]^ Because PEG-IFN-α efficacy may vary from individual to individual, the early recognition of responder patients is important for optimal clinical management. In addition, the identification of the most appropriate therapeutic plan, including optimal treatment timing, is still an open issue. Thus, in order to assess the ability of combination treatments to enhance the therapeutic efficacy in CHB, we performed a retrospective evaluation of the outcomes obtained in HBeAg-positive CHB patients undergoing different therapeutic regimens based on PEG-IFN-α, alone or in combination with NAs.

## 2 Materials and methods

### 2.1 Patients inclusion and exclusion criteria

This retrospective observational study was approved by the ethics committee of the Anhui Medical University, Hefei, China (No. 2012624). At the time of interferon treatment, each patient signed an informed consent, which included the use of relevant data for scientific research and non-commercial purposes.

All chronic HBV-infected HBeAg-positive patients receiving PEG-IFN-α treatment at the First Affiliated Hospital of Anhui Medical University and Second People's Hospital of Fuyang City from 2012 through 2014 were included in this study if they met the following inclusion and exclusion criteria.

The inclusion criteria were as follows: an at least 6-month HBsAg-positivity; negativity for antibodies to hepatitis B e antigen (anti-HBe); a serum alanine aminotransaminase level (ALT) at ≥2 × upper limit of normality (ULN) but ≤10 × ULN and/or at least moderate liver necroinflammation or fibrosis (inflammation and necrosis ≥ grade 2, or fibrosis ≥ grade 2); initial treatment with PEG-IFN-α (PEG-IFN-α-2α, Pegasys, Roche, Shanghai, China or PEG-IFN-α-2b, Introna, Schering-Plough, Shanghai, China), with or without subsequent treatment with NAs (The standard duration of PEG-IFN-α therapy is 48 weeks, and about 30% of HBeAg-positive patients achieve HBeAg seroconversion.^[9]^ For patients who did not have HBeAg seroconversion after 24 weeks of treatment, because there are no internationally reliable drug adjustment criteria, doctors can continue to use PEG-IFN-α, in combination with NA, or replace with NA monotherapy. However, whether, what, and when to change the treatment plan depends on the doctor's experience and the patients’ willingness); completeness of the relevant patient information.

The exclusion criteria were as follows: co-infection with HCV, HIV, or hepatitis delta virus; other liver diseases, including autoimmune hepatitis, alcoholic liver disease, non-alcoholic fatty liver disease, and schistosomiasis; treatment with antiviral drugs for CHB or immunomodulators during the previous 6 months; decompensated cirrhosis, HCC or other malignant tumors; and serious heart, kidney, endocrine, hematopoietic, or psychiatric disorders.

### 2.2 Data collection

Patient characterization data and clinical variables were extracted from the central databases of the two hospitals, which included gender, age, starting time, the use of medication, and the laboratory parameters (including serum ALT, HBsAg, antibodies to hepatitis B surface antigen (anti-HBs), HBeAg, anti-HBe, antibodies to hepatitis B core antigen (anti-HBc), and HBV DNA levels) at baseline, weeks 12, 24, 52, and 76. Serum ALT levels were measured using an automatic biochemical analyzer (Roche, Basel, Switzerland). An ALT level ≤1 × ULN (50 U/L) was considered normal. Serum HBsAg, anti-HBs, HBeAg, anti-HBe, and anti-HBc were tested using commercially available enzyme immunoassays (Abbott Laboratories, North Chicago, IL, USA). The sensitivity of HBsAg assay ranged from 0.05 to 250 IU/mL, some samples with HBsAg >250 IU/mL were not diluted and further tested because of the cost. Serum HBV DNA levels were measured using a TaqMan-based real-time polymerase chain reaction assay (Shanghai ZJ BioTech, Shanghai, China) with a lower detection limit of 1000 copies/mL. HBeAg seroconversion was defined as loss of HBeAg and acquisition of anti-HBe positivity in a previously HBeAg-positive and anti-HBe-negative subject.^[8]^ Patients who did not fulfill this criterion were considered non-responders. The endpoint was HBeAg seroconversion at 76 weeks.

### 2.3 Statistical analysis

The Statistical Program for Social Sciences (SPSS) Version 18.0 (SPSS Inc., Chicago, IL, USA) was used to perform statistical analysis. HBV DNA levels were logarithmically transformed. Quantitative variables were expressed as mean ± standard deviation (SD) for normally distributed data or median (interquartile range) for non-normally distributed data and categorical variables were presented as counts and percentages. The quantitative data were compared using the non-parametric tests and the categorical data were compared using the Chi-squared test. If the sample size of categorical data is small (e.g., the total number of cases <40 or the theoretical frequency <1), the results of the Chi-squared test may be biased. To this end, Fisher's exact test is adopted for analysis. All statistical tests were two-sided and statistical significance was defined as *P* < .05.

## 3 Results

### 3.1 Baseline characteristics

A total of 113 patients, 76 of whom switched to NAs or added NAs therapy (6 changed to ADV combined with LAM, 4 changed to ADV combined with LDT, 24 add ADV, 14 add ETV, 6 add LAM, and 22 add LDT) were included in this study. The duration of PEG-IFN-α treatment was 26 to 260 weeks with a median of 52 weeks. We stratified the patients according to whether or not they experienced HBeAg seroconversion 76 weeks after therapy initiation and their baseline characteristics (including gender, age, combined drug use) are illustrated in Table 1. At 76 weeks after therapy initiation, 44 (38.9%) patients exhibited HBeAg seroconversion (RS group). These patients had lower baseline HBeAg levels than those without HBeAg seroconversion (NRS group) (S/CO = 475.5 vs 751.7; *t* = 2.74; *P* = .007). Both RS and NRS groups did not significantly differ regarding gender, age, ALT, HBV DNA, HBsAg levels at baseline, or the timing of change of treatment regimens (all *P* > .05). However, the group of non-responders comprised a higher proportion of patients with HBsAg levels above 250 IU/mL (68 vs 40; *P* = .05) and displayed higher HBV DNA levels (7.28 vs 6.90; *P* = .07), although these differences were not statistically significant. Finally, the group of non-responders contained a higher proportion of patients who underwent combination therapy with NAs.

**Table 1:**
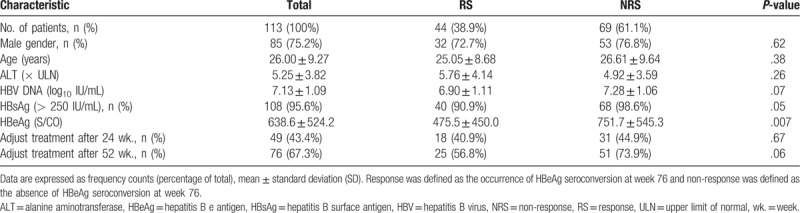
Baseline characteristics of patients with diverse responses at week 76 after therapy.

### 3.2 Quantitative changes in biological markers

**Figure 1:**
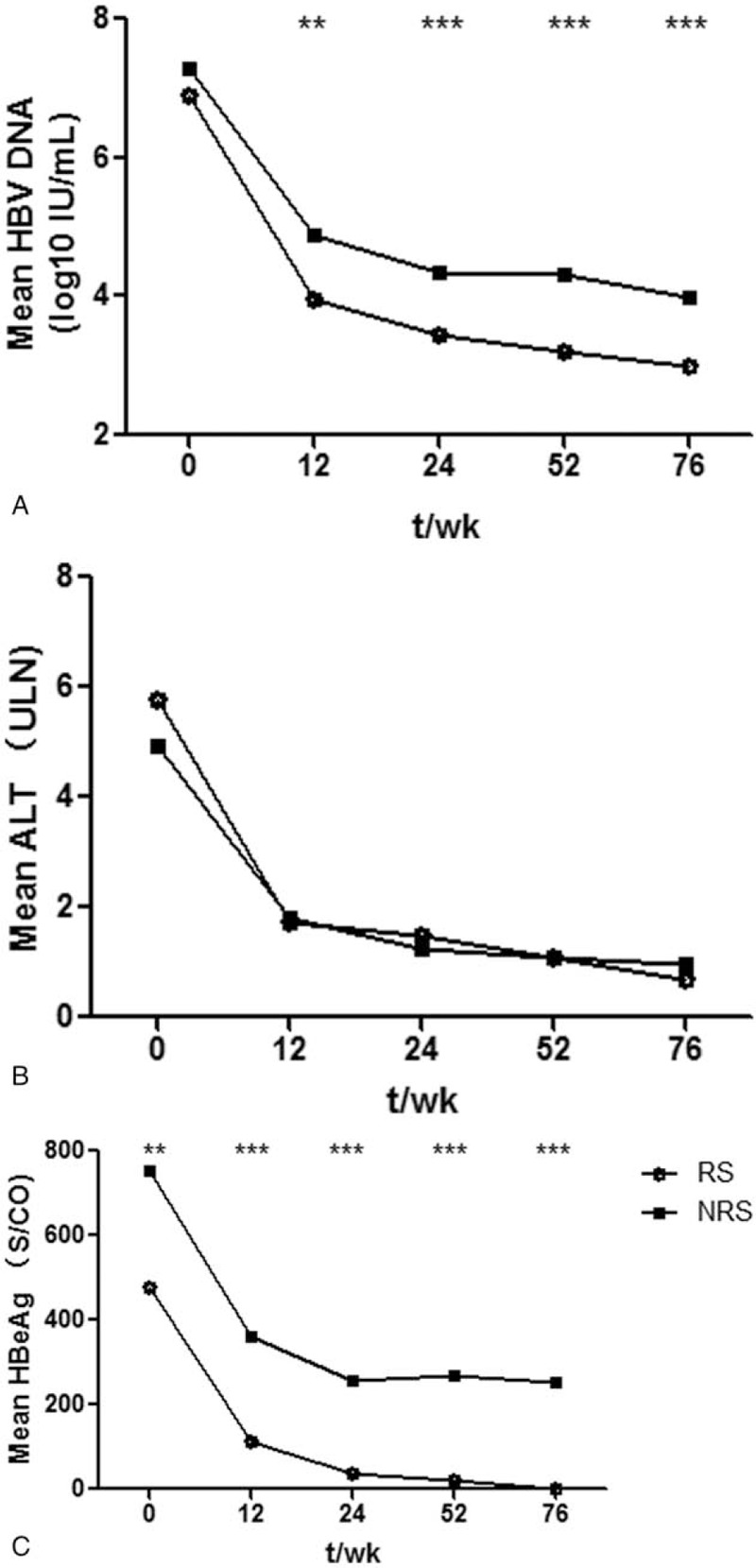
HBV DNA, ALT, and HBeAg levels over time in responders versus non-responders. HBV DNA levels differed very little at baseline in responders and non-responders, but the differences became highly significant during the treatment. ALT levels were not significantly different at baseline or during treatment. HBeAg was significantly different before and after treatment and, although both groups had significant declines during the first 12 weeks, almost no decrease was observed in the non-responders after 24 weeks. Responders were defined as loss of HBeAg and acquisition of anti-HBe positivity at 76 weeks in CHB patients who were positive for HBeAg before PEG-IFN-α treatment. Patients who did not fulfill this criterion was considered non-responders. ALT = alanine aminotransferase, 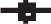 RS = responders, 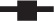 NRS = non-responders, HBeAg = hepatitis B e antigen, HBV = hepatitis B virus. ∗*P* < .05, ∗∗*P* < .01, and ∗∗∗*P* < .001.

The mean levels of HBV DNA, ALT, and HBeAg over time in patients receiving PEG-IFN-α, with or without NAs therapy, are displayed in Fig. 1. Among the 44 patients experiencing HBeAg seroconversion, the levels of HBV DNA differed very little at baseline and decreased more rapidly compared to HBeAg non-responders, with statistically significant differences at weeks 24, 52, and 76 (all *P* < .001). ALT levels were not significantly different between responders and non-responders groups at baseline or during treatment (all *P* > .05), and there was an intersection between the ALT profiles of the two groups. In the responders, HBeAg decreased consistently during treatment, while the non-responders showed a significant decrease in the first 24 weeks, but it rebounded slightly at 52 weeks compared to 24 weeks, and then decreased again at 76 weeks. HBV DNA exhibited a similar pattern.

### 3.3 Predictive value of seroconversion indicators

**Figure 2:**
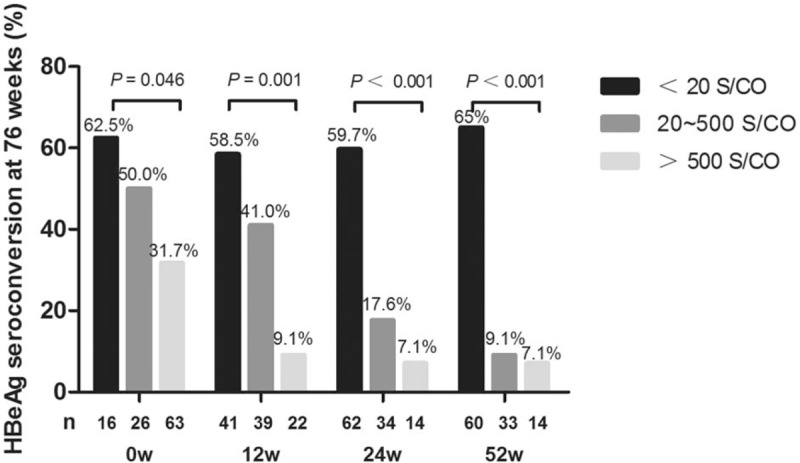
HBeAg as a predictor of sustained serological responses. HBeAg levels were grouped according to cut-off values of 500 and 20 S/CO. HBeAg was found to be a predictor of serological conversion at baseline and at 12, 24, and 52 weeks. If HBeAg > 500 S/CO at 24 weeks or > 20 S/CO at 52 weeks, HBeAg seroconversion at the end of follow-up was unlikely. When HBeAg was reduced below 20 S/CO, there was a high probability of HBeAg seroconversion. Serological response was defined as the occurrence of HBeAg seroconversion at 76 weeks. The Chi-squared test was used in this analysis. = HBeAg < 20 S/CO, 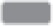 = HBeAg 20–500 S/CO, 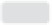 = HBeAg > 500 S/CO, HBeAg = hepatitis B e antigen.

ALT, HBeAg, and HBV DNA were subgrouped according to previous studies and their ability to predict the response to treatment was evaluated.^[18,19]^ As shown in Table 2, ALT and HBV DNA levels were not statistically different between responders and non-responders (*P* > .05). Notably, the incidence of HBeAg seroconversion was significantly higher among patients with HBeAg S/CO ≤ 500 (OR = 2.60, 95% CI: 1.16–5.83, χ^2^ = 5.52, *P* = .02, PPV = 54.76%, NPV = 68.25%) at baseline, with HBeAg S/CO ≤ 20 (OR = 3.37, 95% CI: 1.47–7.73, χ^2^ = 8.53, *P* = .003, PPV = 58.54%, NPV = 70.49%) or a higher than 10-fold HBeAg decline (OR = 3.55, 95% CI: 1.50–8.37, χ^2^ = 8.67, *P* = .003, PPV = 62.16%, NPV = 68.33%) at 12 weeks, or with HBeAg S/CO ≤ 15 (OR = 10.35, 95% CI: 4.09–26.20, χ^2^ = 28.03, *P* < .001, PPV = 64.29%, NPV = 85.19%) at 24 weeks. Thus, patient classification based on the level of HBeAg had a high predictive value for treatment response. The results clearly showed that the HBeAg level at week 12, 24, and 52 was a predictor of serological conversion (all *P* < .001) (Fig. 2). If HBeAg S/CO was higher than 500 at week 24 or higher than 20 at week 52, HBeAg seroconversion at the end of follow-up was unlikely. On the other hand, if at week 52 HBeAg S/CO declined below 20, seroconversion was highly probable.

**Table 2:**
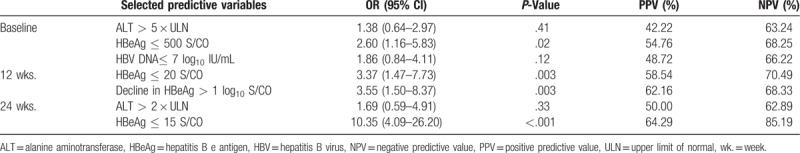
The predictive value of relevant indicators at baseline and week 12, and 24 influencing hepatitis B e antigen seroconversion at week 76.

### 3.4 Changes in the biological markers of patients without HBeAg seroconversion at week 24

**Figure 3:**
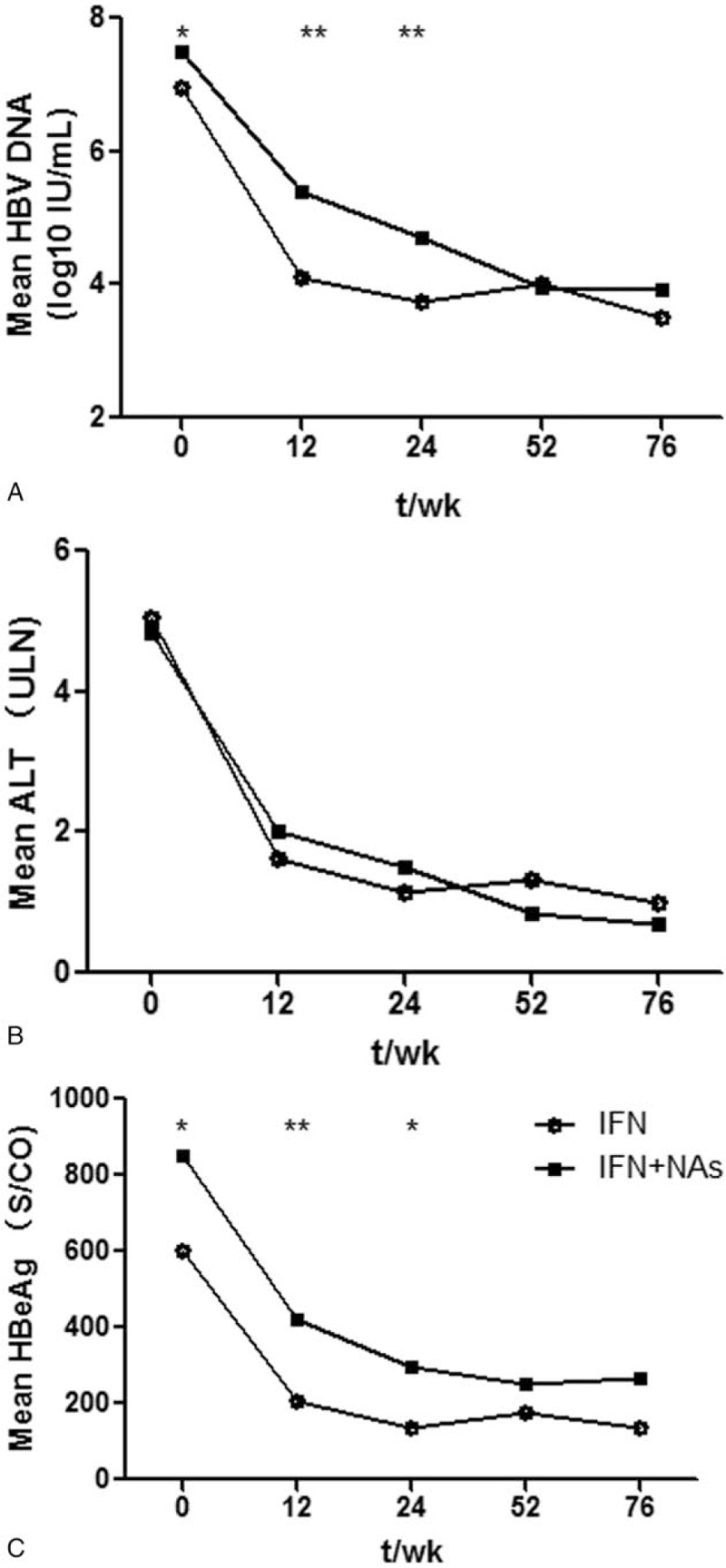
Changes in biological markers of patients without HBeAg seroconversion at 24 weeks. Patients who did not exhibit HBeAg seroconversion at 24 weeks were divided into two groups according to whether they were received combined therapy or not. HBV DNA and HBeAg were significantly different between the two groups at baseline and at 12 and 24 weeks. ALT was not significantly different either at baseline or during treatment. ALT = alanine aminotransferase, 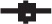 = IFN monotherapy, 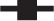 = combination therapy with IFN plus NAs, HBeAg = hepatitis B e antigen, HBV = hepatitis B virus. ∗*P* < .05 and ∗∗*P* < .01.

Patients who did not exhibit HBeAg seroconversion at week 24 were divided into two groups receiving combined treatment with NAs or continuing PEG-IFN-α monotherapy, respectively. HBV DNA and HBeAg in patients without HBeAg seroconversion at week 24 were significantly higher at baseline, weeks 12 and 24 (all *P* < .05) (Fig. 3). Patients who did not have HBeAg seroconversion at 24 weeks were more likely to be treated with a combination of PEG-IFN-α and NAs if their HBV DNA and/or HBeAg were still high, while patients with lower HBV DNA and HBeAg were more likely to continue PEG-IFN-α monotherapy. Interestingly, after the IFN/NA combination therapy, HBV DNA and HBeAg levels were significantly reduced, and there was no statistically significant difference between the two groups at 52 or 76 weeks (all *P* > .05). Although HBV DNA and/or HBeAg were higher at baseline and at week 24 in the group receiving the combined therapy, compared to the responders maintained on monotherapy, at week 76 the rate of HBeAg seroconversion was similar in the two groups (24% vs 26.8%, *P* = .76; Table 3).

**Table 3:**
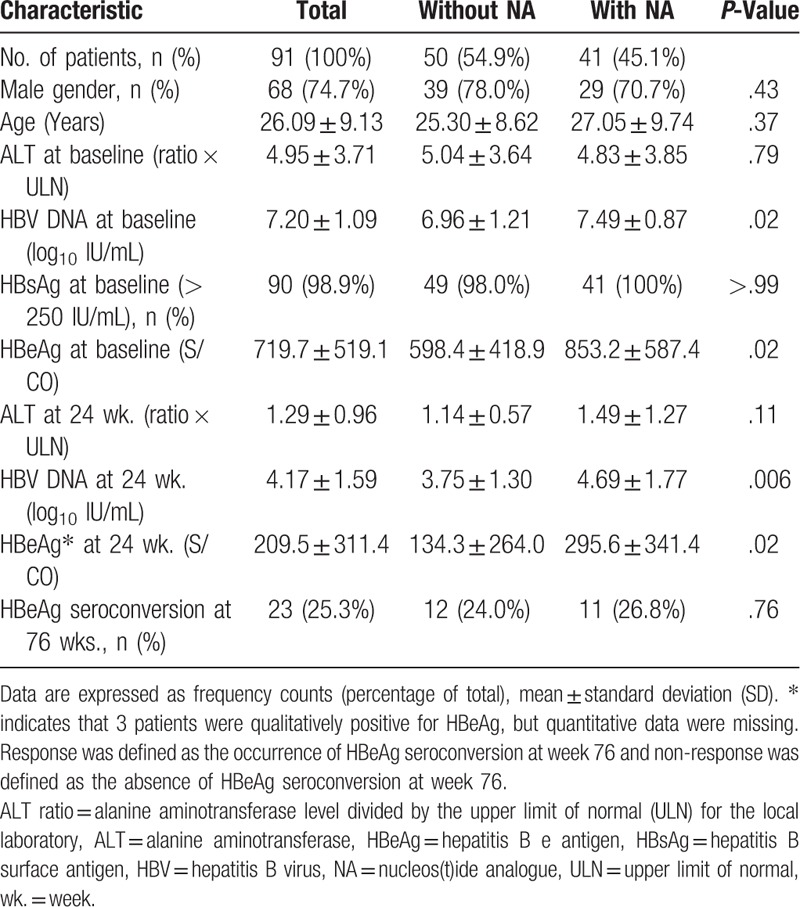
Baseline status of non-responders with or without NA at week 24.

### 3.5 Response of patients without HBeAg seroconversion to NA-based combination therapy

**Figure 4:**
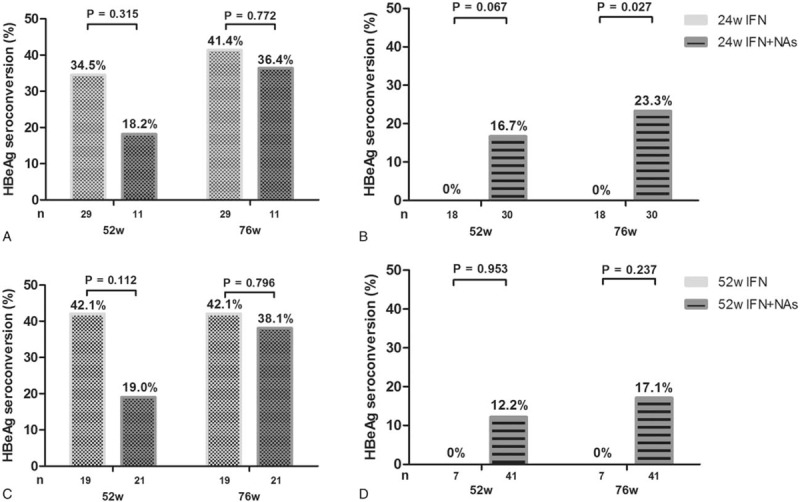
Serologic response to combined therapy in patients without HBeAg seroconversion at 24 weeks. Response to combined IFN/NA therapy in (A) patients with HBeAg S/CO between 1 and 20 at 24 weeks, (B) patients with HBeAg S/CO > 20 at 24 weeks, (C) patients with HBeAg S/CO between 1 and 20 at 52 weeks, and (D) patients with HBeAg S/CO > 20 at 52 weeks.  = continued interferon monotherapy, 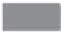 = treated with a combination of IFN and NAs at 24 weeks, 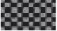 = HBeAg S/CO between 1 and 20 at 24 weeks, 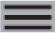 = HBeAg S/CO > 20 at 24 weeks.

Patients who did not exhibit HBeAg seroconversion at 24 weeks were stratified on the basis of their HBeAg levels at 24 weeks of treatment and divided into groups according to the time of NA addition (Fig. 4). In patients with HBeAg S/CO between 1 and 20 at week 24, continued IFN treatment often led to HBeAg seroconversion, while in patients with an HBeAg S/CO > 20, continued IFN treatment did not result in significant HBeAg seroconversion. In the latter patients, NA-based combination therapy induced remarkably higher seroconversion rates, compared to patients with similar HBeAg profiles who were maintained on interferon monotherapy (*P* = .03, Fig. 4B). However, the combined therapy was ineffective in patients exhibiting HBeAg S/CO > 20 at week 52 (both *P* > .05, Fig. 4D). Finally, the administration of NAs, from week 24 or 52, to patients exhibiting HBeAg S/CO between 1 and 20 at week 24 did not significantly improve the therapeutic efficacy (all *P* > .05, Fig. 4A and C).

## 4 Discussion

Antiviral therapy can reduce the risk of end-stage liver disease (liver failure, decompensated cirrhosis, and HCC). Current antiviral therapies include NAs or IFN monotherapy and combination therapy. However, monotherapies have failed to achieve short-term HBeAg seroconversion and/or HBsAg elimination in most CHB patients,^[8]^ whereas various studies have shown that combination therapies may result in better efficacy.^[15–17]^ However, the latter approach has intrinsic disadvantages, the most important being a high economic burden and the occurrence of adverse events. Therefore, it is important to identify the patients who may benefit from combination therapy and define the optimal treatment timing.

Since PEG-IFN-α has a limited treatment duration, a higher rate of HBeAg and HBsAg seroconversion, a higher chance of sustained off-treatment virologic response, and no associated drug resistance.^[20]^ The Asian Pacific Association for the Study of the Liver (APASL), the American Association for the Study of Liver Diseases (AASLD), and EASL recommend PEG-IFN-α as a first-line treatment for CHB patients with moderately replicating HBV and early liver disease.^[5,8,9]^ However, PEG-IFN-α efficacy may vary from individual to individual and only about 30% of HBeAg-positive CHB patients achieve HBeAg seroconversion.^[9]^ Therefore, it is important to distinguish, as early as possible, patients with a high probability of achieving HBeAg seroconversion from non-responders. In this study, we also showed that both the starting HBeAg level and the extent of the initial HBeAg decline in patients under PEG-IFN treatment were strong predictors of therapeutic response. In particular, patients with HBeAg S/CO ≤ 500 at baseline or ≤20 at week 12 and with a >10-fold HBeAg decrease at week 12 tended to display a sustained response to the PEG-IFN-based treatment, consistently with previous studies.^[19,21]^ Importantly, combined NAs/PEG-IFN therapy increased the therapeutic efficacy in patients displaying an HBeAg S/CO > 20 after 24 weeks of PEG-IFN treatment.

According to current treatment recommendations, HBV DNA, ALT and HBsAg levels, HBV genotype, and high activity scores on liver biopsy should be considered as predictors of response to IFN-based therapies in HBeAg-positive CHB patients.^[9]^ In this study, we confirmed that HBeAg levels and the extent of HBeAg decline at specific treatment stages were closely related to the rate of response for PEG-IFN-based treatment (Table 2). The incidence of HBeAg seroconversion was significantly higher among patients with HBeAg S/CO ≤ 500 at baseline, HBeAg S/CO ≤ 20 and a higher than 10-fold HBeAg drop at week 12, or HBeAg S/CO ≤ 15 at week 24. If HBeAg S/CO was higher than 500 at week 24 or higher than 20 at week 52, HBeAg seroconversion at the end of follow-up was unlikely. Consistently, a recent large study focusing on PEG-IFN-α therapy demonstrated that HBeAg seroconversion was significantly dependent on pre-treatment HBeAg levels and high initial levels of HBeAg resulted in low rates of HBeAg seroconversion.^[21]^ This may be due to impaired effector function of T cells. Ye et al found that the effector function of T cells is impaired during chronic HBV infection and that increases in viral load or antigen levels were associated with enhanced expression of co-inhibitory receptors on the surface of exhausted T cells.^[22]^ It is known that, in addition to activating the antiviral mechanisms of infected cells, IFN can promote the maturation of dendritic cells, thus facilitating CD4+ T cell differentiation into either Th1 or Th2 cells. Moreover, pathogen-experienced antigen presenting cells are capable of cross-presentation and stimulate CD8+ T cell clonal expansion and proliferation.^[23]^ When T cell function is damaged, IFN does not effectively stimulate the immune responses, resulting in poor efficacy. A lack of (or insufficient) HBeAg decrease after 24 weeks likely reflected an IFN failure to stimulate host immunomodulation. However, the addition of NAs at week 24 increased HBeAg seroconversion in non-responders with HBeAg S/CO > 20. This was likely due to a restoration of T cell function by combination therapy, in addition to a stronger on-treatment HBV DNA suppression.^[24]^

The group of non-responders contained a higher proportion of patients who underwent combination therapy with NAs. However, this could be due to a bias in patient selection, since combination therapy may have been especially assigned to patients with more severe disease or who were unresponsive to previous courses of PEG-IFN-α monotherapy. In this study, the HBeAg seroconversion rate at 76 weeks could still be improved by NAs combination in patients with high levels of HBeAg (HBeAg S/CO > 20) after 24 weeks of interferon treatment, indicating that combined treatment may still improve the efficacy in patients poorly responding to interferon alone. Therefore, it is necessary to further explore the efficacy of interferon-NAs combination therapy in this subset of patients.

This study is a retrospective analysis of data obtained from two clinical centers and is characterized by limited sample size. The evaluation of larger sample populations with longer follow-ups is required to establish optimal therapeutic regimens. In this study, a very small number of patients used interferon for more than 52 weeks, or even up to 260 weeks, which was mainly because the quantitative reduction of HBeAg and HBsAg was very obvious after the use of interferon in these patients. Some patients expected to achieve functional cure (disappearance of HBsAg or serum conversion) and insisted on continued treatment with interferon.

In conclusion, this study suggested that both the HBeAg level at specific treatment stages and the extent of HBeAg decline after treatment initiation were predictors of HBeAg seroconversion at week 76 in HBeAg-positive patients receiving PEG-IFN-based treatment. In addition, combined NAs/Peg-IFN therapy proved to be a valuable option for CHB patients displaying relatively high levels of HBeAg after 24 weeks of treatment with PEG-IFN-α.

## Author contributions

**Conceptualization:** Zhenhua Zhang, Jiabin Li.

**Data curation:** Yafei Zhang, Wei Li, Zhongping Liu, Jun Ye, Guizhou Zou.

**Formal analysis:** Yafei Zhang, Wei Li, Zhongping Liu, Jun Ye, Guizhou Zou.

**Funding acquisition:** Zhenhua Zhang, Jiabin Li.

**Investigation:** Yafei Zhang, Wei Li, Jun Ye, Guizhou Zou.

**Methodology:** Yafei Zhang.

**Project administration:** Zhenhua Zhang, Jiabin Li.

**Supervision:** Zhenhua Zhang, Jiabin Li.

**Writing – original draft:** Zhenhua Zhang, Yafei Zhang, Zhongping Liu.

**Writing – review & editing:** Jun Ye, Guizhou Zou, Zhenhua Zhang, Jiabin Li.
